# Some Nerve Diseases

**Published:** 1890-11-08

**Authors:** 


### THE LONDON POST-GRADUATE COURSE AT THE PADDINGTON INFIRMARY.

### SOME NERVE DISEASES.

Dr. Bristowe, in his lecture, first described and showed some obscure cases of nervous diseases he had brought from St. Thomas's Hospital, and then some patients who were in the infirmary. The first case was a man aged 26, who, at the time being, was quite well, a slight tremor of the head and then of the right arm came on. Later his speech became affected, but he continued at his occupation as a policeman till a fortnight before admission into St. Thomas's Hospital two months ago. On admission there was marked tremor of the head, neck, and right arm, with some difficulty of speech and of walking. At the present time the tremor of the head ceased when the part was at rest, but was aggravated on movement, much as in disseminated sclerosis; there was also tremor in the right arm, and difficulty of walking, especially in the right leg; the tendon reflexes were very variable and frequently absent, there was slight nystagmus and double vision, the speech as in general paralysis of the insane was much affected, together with very well marked tremor of the lips. The pupils were equal and reacted to light readily. It was very difficult to say what was the nature of the disease, if it was a case of disseminated sclerosis it was only right sided, and the character of the speech was not typical. It might be a case of general paralysis of the insane in which the paralytic symptoms had come on before the mental ones, on the other hand it might be a case of myelitis with sub-acute characters. The second case was that of a man who was originally admitted into a surgical ward some two years ago suffering from weakness in the right arm and pain between the shoulders, supposed to be due to an aneurysm. The history was, that for a few weeks he had suffered severe pain between the shoulders, which extended down the left arm; the pain then passed from this arm and went down the right one; this was shortly afterwards followed by paralysis of the right arm and a rapid wasting of the muscles, and the hand soon presented the glossy-skinned appearance. There was, however, reason to believe that it was of a specific nature, and so he was put on mercury and iodide of potassium. Under this treatment he much improved, and it was then noticed that the left side of the face was somewhat weak, and the tongue when protruded tended to the right, and the posterior pillar of the fauces of the left side moved less than that of the right. All the signs had much improved, and the arm was now rapidly recovering, most of the movements having already returned. It was probably a case of scattered disease of the membranes; in fact, specific disease in some form affecting the membranes of the cord in the first or second cervical region. It was more probably disease of the membranes than of the cord itself from the amount of pain he had suffered; and there having been no affection of the legs it was probably not one of myelitis. It would appear that the nerves had become involved in a local disease affecting the membranes, probably of an inflammatory rather than of a gummatous nature. The third case was that of a man aged 22, who had an injury to the head nine years ago; he was insensible for two days, but afterwards completely recovered. During the last twelve months he had suffered from giddiness and staggering with sensations at the back of his neck, and a tendency to arch the head backwards; these attacks come on irregularly, and last from a few minutes to sometimes as long as half-an-hour. They appear to be of an epileptiform nature. He had also a central scotoma in both eyes, with well marked optic atrophy, but the yellow spots were healthy. The appearance of the scotoma was not like tobacco poisoning, and there was no colour blindness. There was no mark of the original head injury and he had completely recovered from this two years before the present symptoms came on, but it might be suspected that the injury had something to do with his symptoms. The affection of the eyes could not be explained by a peripheral, but by a central lesion. Thus it might be connected with the original injury or it might be a case of syphilitic disease of the brain, but there was no history of this to be ascertained. The fourth case was that of a man forty years old, a dustman, who had had good health. Fourteen years ago he had received a blow on the left side of the head from a poker, after which he was insensible for forty-eight hours. He recovered from this and remained well for two years, when epileptic fits ensued, which were very severe and occurred in groups. He was in the hospital seven years ago, and was then trephined over the left side of the head, the dura mater was not opened, and there was no evidence of a fracture of the bone. For the next two years the fits were less frequent and less severe; after this period, the fits becoming more severe, he was admitted into the infirmary, and Mr. Horsley trephined and opened the dura mater and removed an old cicatrix, probably the seat of an old hæmorrhage produced by the blow a few years before. After the second operation the fits again improved, but he became hemiplegic. He also had aphasia after the operation, but this had improved of late. He cannot read or write, but can name some letters. He cannot write from a copy or from dictation, but he can write his name with the left hand. He has also complete right hemianopsia, which was observed some two years ago. The right arm has become paralysed, wasted, and contracted, presenting more the appearance of progressive muscular atrophy than from true brain disease. It is probable that now it is a case of descending degeneration of the motor tract from the surface of the brain, and that the neuclei in the cord have become affected, and hence the paralysis of the arm. The fits always begin in the right big toe and then extend along the right arm, and after this the left side is occasionally involved. The fourth case was that of a man aged fifty-five, a navvy, who had enjoyed good health, but had been rather a free drinker. Two years ago some earth fell upon him and he was stunned, remaining insensible for some time. He recovered, and was quite well till the 7th of October last, when he had a fit, which came on in the night, with loss of consciousness. A week later he had another fit, which also came on in the night, and this was followed by weakness in the left leg, which presented much the condition of spastic paralysis. The leg was stiff, the tendon reflexes were exaggerated, and there was marked ankle clonus, and he had attacks of spinal apoplexy. Thus, as in the previous case, this might be one of degeneration passing down the cord from the brain.

